# RAGA: a reference-assisted genome assembly tool for efficient population-scale assembly

**DOI:** 10.1093/hr/uhaf207

**Published:** 2025-08-11

**Authors:** Ru-Peng Zhao, Yu-Hong Luo, Wen-Zhao Xie, Zu-Wen Zhou, Yong-Qing Qian, Si-Long Yuan, Dong-Ao Li, Jiana Li, Kun Lu, Xingtan Zhang, Jia-Ming Song, Ling-Ling Chen

**Affiliations:** State Key Laboratory for Conservation and Utilization of Subtropical Agro-Bioresources, College of Life Science and Technology, Guangxi University, Nanning 530004, China; State Key Laboratory for Conservation and Utilization of Subtropical Agro-Bioresources, College of Life Science and Technology, Guangxi University, Nanning 530004, China; Ministry of Education Key Laboratory of Molecular and Cellular Biology, Hebei Research Center of the Basic Discipline of Cell Biology, College of Life Sciences, Hebei Normal University, Shijiazhuang 050024, China; State Key Laboratory for Conservation and Utilization of Subtropical Agro-Bioresources, College of Life Science and Technology, Guangxi University, Nanning 530004, China; College of Agronomy and Biotechnology, Southwest University, Chongqing 400715, China; State Key Laboratory for Conservation and Utilization of Subtropical Agro-Bioresources, College of Life Science and Technology, Guangxi University, Nanning 530004, China; State Key Laboratory for Conservation and Utilization of Subtropical Agro-Bioresources, College of Life Science and Technology, Guangxi University, Nanning 530004, China; College of Agronomy and Biotechnology, Southwest University, Chongqing 400715, China; College of Agronomy and Biotechnology, Southwest University, Chongqing 400715, China; National Key Laboratory for Tropical Crop Breeding, Shenzhen Branch, Guangdong Laboratory for Lingnan Modern Agriculture, Genome Analysis Laboratory of the Ministry of Agriculture, Agricultural Genomics Institute at Shenzhen, Chinese Academy of Agricultural Sciences, Shenzhen, Guangzhou 518120, China; College of Agronomy and Biotechnology, Southwest University, Chongqing 400715, China; State Key Laboratory for Conservation and Utilization of Subtropical Agro-Bioresources, College of Life Science and Technology, Guangxi University, Nanning 530004, China; Yazhouwan National Laboratory, Sanya 572025, China

## Abstract

High-quality reference genomes at the population scale are fundamental for advancing pan-genomic research. However, high-quality genome assembly at the population scale is costly and time-consuming. To overcome these limitations, we developed Reference-Assisted Genome Assembly (RAGA), a hybrid computational tool that combines *de novo* and reference-based assembly approaches. RAGA efficiently employs existing reference genomes from the same or closely related species in combination with PacBio HiFi reads to produce high-quality alternative long sequences. These sequences can be integrated with *de novo* assemblies to improve assembly quality across population-scale datasets. The performance of RAGA across various plant genomes demonstrated its ability to reduce the number of contigs, decrease gaps, and correct genome assembly errors. The implementation of RAGA (available at https://github.com/wzxie/RAGA) significantly streamlines population-scale genome assembly workflows, providing a robust foundation for comprehensive pan-genomic investigations. This tool represents a substantial advancement in making large-scale genomic studies more accessible and efficient.

## Background

The pan-genomic era has brought an urgent demand for population-scale genome assemblies to comprehensively characterize genetic variation, elucidate species diversity, and unravel evolutionary mechanisms [1, 2]. While high-quality genome assemblies are recognized as foundational for pan-genomic research, as demonstrated in human pan-genome studies [3], only a limited number of plant species, such as the *Citrullus* genus, have achieved telomere-to-telomere (T2T) super pan-genomes, exemplified by the integration of 27 complete watermelon genomes [4]. With the continuous improvement of plant genome assemblies and databases such as plantGIR [5], TGDF [6], and SoIR [7], an unavoidable pan-genomic era has arrived, which demands parallel advancements in assembly workflows.

Recent advances in hybrid assembly strategies, combining ultra-long Oxford Nanopore (ONT) reads with highly accurate PacBio HiFi data using tools like verkko [8] and hifiasm (UL) [9], have enabled *de novo* T2T assembly for numerous genomes. However, applying such resource-intensive sequencing approaches to all samples in population-scale studies remains prohibitively expensive [10]. Consequently, achieving high-quality genome assemblies at scale continues to pose a significant challenge. Although existing tools such as DEGAP [11], TGS-gapcloser [12], and Nextpolish2 [13] offer optimization capabilities, their application across large and diverse datasets often involves complex, sample-specific adjustments and limiting scalability. To address these limitations, employing high-quality reference genomes has emerged as a powerful strategy for guiding and refining target genome assemblies [14]. Tools like RagTag [15] and quarTeT [16] utilize collinearity between reference genomes and target contigs to improve scaffolding, a method successfully employed in constructing the pan-genomes of *Arabidopsis thaliana* [17, 18] and rice [19].

In this study, we systematically evaluate the role of reference genomes in optimizing *de novo* assembly and introduce Reference-Assisted Genome Assembly (RAGA), a new tool designed to enhance assembly quality. By exploiting synteny between a reference genome and the target assembly, along with PacBio HiFi reads from the target genome, RAGA generates alternative long sequences that emulate Oxford Nanopore Technology (ONT) ultra-long reads for *de novo* assembly. Extensive validation across phylogenetically diverse species demonstrates that integrating these sequences with original HiFi reads significantly improves assembly contiguity and accuracy while avoiding reference-derived biases. Current genome assembly workflows often require multiple rounds of polishing and gap-filling to address assembly faults. RAGA resolves these limitations by improving initial *de novo* assembly quality, thereby reducing the complexity of downstream corrections and facilitating more efficient gap closure. We anticipate that RAGA will substantially lower the barriers to generating complete, high-quality genome assemblies at the population scale, providing robust genomic resources for future pan-genomic research.

## Results

### The construction of RAGA

**Figure 1:**
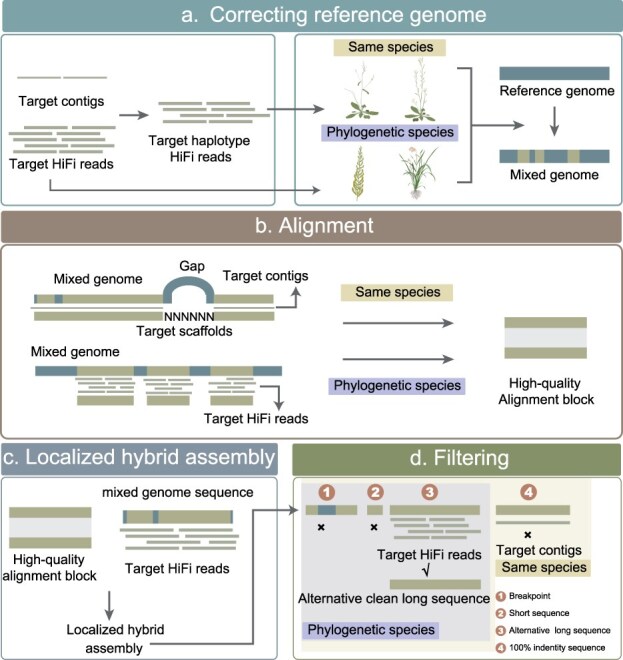
The construction pipeline of RAGA. (a) RAGA corrects the reference genome using target HiFi reads to produce a mixed genome that closely resembles the target genome. (b) Different alignment processes are designed for various types of reference genomes to obtain high-quality alignment blocks between the mixed genome and the target genome. (c) Each high-quality alignment block undergoes localized hybrid assembly to obtain raw alternative long sequences. (d) The obtained raw long sequences are filtered to obtain clean alternative long sequences.

The RAGA framework employs a reference-guided approach to enhance contig assembly while minimizing reference-derived artifacts. Our methodology features a four-stage pipeline: (i) reference genome correction, (ii) sequence alignment, (iii) localized hybrid assembly, and (iv) rigorous quality filtering (Fig. 1).

In the initial process, RAGA performs reference genome polishing using target-derived HiFi reads, generating an optimized hybrid reference that better represents the target genome’s characteristics (Fig. 1a). This corrected reference serves as the foundation for subsequent analyses. When the reference genome and the target assembly originate from the same species, RAGA utilizes the mixed genome as a reference to perform scaffolding and alignment on the target contigs. Through rigorous screening, RAGA retains high-quality alignment blocks that flank scaffold gaps in the target scaffolds, optimizing computational resource allocation. However, when using phylogenetically distant specie as reference genome, the collinearity between reference and target genomes may be highly complex and disordered, making it difficult to predict gap regions in the target genome assembly. In such cases, RAGA directly aligned target HiFi reads to the hybrid reference to identify high-quality alignment blocks (Fig. 1b), followed by localized hybrid assembly of each qualified block using hifiasm (UL) [9] (Fig. 1c). To ensure assembly fidelity, RAGA implements a multistage quality control pipeline, including (i) eliminates sequences with HiFi read coverage breakpoints, (ii) discards sequences showing either complete identity or excessive divergence from the hybrid assembly, (iii) performs length-based selection to retain only the most robust alternative long sequences (Fig. 1d). This comprehensive quality assurance protocol guarantees the production of highly accurate alternative sequences while effectively preventing the introduction of reference-derived artifacts.

Our results demonstrate that RAGA-generated sequences can extend into previously misassembled genomic regions. When these sequences are incorporated into the RAGA hybrid assembly, they can resolve previous assembly errors, thereby improving the quality of target genome assembly (Fig. S1). The RAGA methodology distinguishes itself from conventional polishing tools through its unique integration strategy, combining RAGA-derived sequences with original sequencing data (PacBio HiFi and Hi-C reads) for comprehensive hybrid assembly (Fig. S2). This approach provides fundamental improvements to *de novo* assembly rather than merely patching existing assemblies, representing a paradigm shift in reference-assisted genome assembly.

### The performance of RAGA in *Arabidopsis* population

To assess the performance of RAGA for population-scale *de novo* assembly, we applied it to the pan-genome of the model plant *A. thaliana*. Utilizing PacBio HiFi sequencing data from 80 *Arabidopsis* accessions obtained from two published pan-genome studies [17, 18], we implemented RAGA to refine individual genome assemblies by employing three T2T reference genomes of *A. thaliana* (Col-0, Col-CEN, and Col-PEK) [20–22].

**Figure 2:**
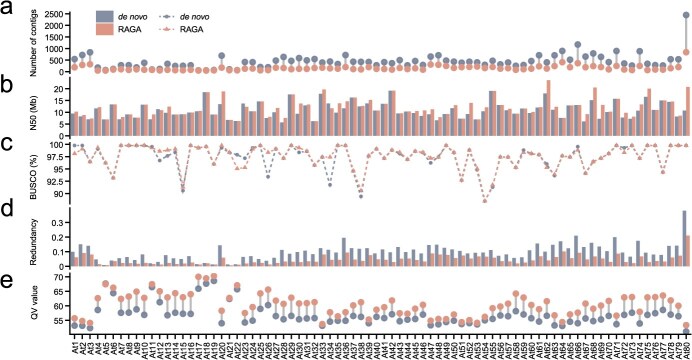
Statistics of assembly indicators for *A. thaliana* population, including *de novo* assembly and RAGA-optimized assembly. (a) Lollipop diagram represents the number of contigs between RAGA-optimized and *de novo* assemblies. (b) Bar chart represents N50 length of contigs between RAGA-optimized and *de novo* assemblies. (c) Line chart represents BUSCO completeness between RAGA-optimized and *de novo* assemblies. (d) Bar chart represents redundancy ratios between RAGA-optimized and *de novo* assemblies. (e) Lollipop diagram represents QV scores between RAGA-optimized and *de novo* assemblies.

RAGA reduced the number of contigs in all assemblies, ranging from 7 to 1606 contigs, and the average reduction is 293 contigs per assembly (Fig. 2a). It increased the average contig N50 in 54 assemblies (67.5% of the total) by ~1.66 Mb (Fig. 2b). Notably, RAGA-optimized assemblies exhibited 75 fewer assembly gaps and three additional gap-free chromosomes compared to *de novo* assemblies (Fig. S3a and b). These findings demonstrate that RAGA significantly enhances assembly continuity in most cases. There were no significant differences between the RAGA-optimized and *de novo* assemblies in the Benchmarking Universal Single-Copy Orthologs (BUSCO) [23] assessment and k-mer-based evaluation (Fig. 2c; Fig. S3c–e), except for samples AT26 and AT34, where BUSCO completeness improved by 4.94% and 5.64%, respectively, after RAGA optimization. RAGA-optimized assemblies showed closer concordance with reference genome sizes (Fig. S3f), and demonstrated 5.38% lower redundancy on average compared to *de novo* assemblies (Fig. 2d). These findings indicate that RAGA can effectively reduce genome redundancy and improve genome completeness. RAGA enhanced quality value (QV) in 79 of 80 assemblies (Fig. 2e), with QV improvements ranging from 0.32 to 6.78 (mean improvement of 3.71). Furthermore, the regional assembly quality indicator (R-AQI) and structural assembly quality indicator (S-AQI) of RAGA-optimized assemblies were comparable to those of *de novo* assemblies (Fig. S3g and h). The CEN180 sequences are a crucial component of Arabidopsis centromeres [21]. Studies revealed that *de novo* assemblies and RAGA-optimized assemblies exhibited comparable total amounts of CEN180 sequences, with no significant difference observed (Fig. S3i). However, compared to *de novo* assemblies, a greater proportion of CEN180 sequences in RAGA-optimized assemblies were located in contigs longer than 1 Mb (Fig. S3j), demonstrating that RAGA enhanced centromere continuity in the *Arabidopsis* population. These comprehensive results demonstrate RAGA’s ability to enhance multiple aspects of assembly quality while maintaining biological accuracy in population-scale genomic studies.

We conducted comprehensive single nucleotide polymorphism (SNP) analyses to evaluate potential allele introgression from reference genomes (Col-0, Col-CEN, and Col-PEK [17–19]) during RAGA optimization, employing both read-based and assembly-based approaches. Using read-based SNP calling as a gold standard, we compared recall, precision, and F1 scores between *de novo* and RAGA-optimized assemblies. Statistical analysis showed no significant difference (*P* > 0.05) in these metrics (Fig. S4), indicating that RAGA does not introduce reference alleles that could bias population SNP analyses (Complete statistical results, including *P*-values and 95% confidence intervals for all *A. thaliana* population analyses, are available in Table S1).

Detailed examination of the assembly composition revealed that all contigs in RAGA-optimized assemblies were exclusively constructed from original HiFi reads, with the alternative long sequences serving only as guides during assembly (Table S2). This critical finding demonstrates that despite their role in guiding assembly, these reference-derived sequences do not physically incorporate into the final contigs. The stringent quality filters applied during alternative sequence generation, combined with this inherent preservation of original read composition, ensure that RAGA maintains assembly fidelity without introducing reference-derived artifacts. These results collectively confirm that RAGA optimization preserves the genetic integrity of the target genome while improving assembly quality.

### The performance of RAGA in rice genome assembly

Our comprehensive evaluation of *A. thaliana* population established RAGA’s effectiveness in enhancing population-scale genome assemblies, with an average PacBio HiFi sequencing depth of 41× (Table S3). To further investigate RAGA’s performance with high-depth sequencing data, we applied the method to several rice cultivars (Table S4).

For the *indica* rice cultivar MH63 [24], RAGA generated ~50 Mb of alternative long sequences using multiple reference genomes (T2T-TIP [25], ZS97, HuaZhan, J4155S, LK638S, and XL628S [26]). Subsequent hybrid assembly with PacBio HiFi reads produced a significantly improved MH63 genome assembly. The RAGA-optimized assembly demonstrated superior continuity metrics, including 426 fewer contigs, 250-kb increase in contig N50, closure of four genomic gaps, and resulting in a complete gap-free assembly. Quality assessments also revealed comprehensive improvements, including base-level accuracy QV increase by 1.28 and regional and structural quality indices (R-AQI and S-AQI) increase by 0.13 and 0.25, respectively. Finally, the genome size of RAGA-optimized assembly is close to the 396-Mb MH63 T2T genome and smaller than the *de novo* assembly, indicating that RAGA reduces redundancy in MH63 assembly (Table 1).

**Table 1:** Genome assembly metrics using RAGA on multiple species with PacBio HiFi sequencing data.

Species variety	Assembly process	Total contig length (Mb)	Contig number	N50 length (Mb)	Gap number	QV	Completeness (%)	R-AQI	S-AQI
Rice (MH63)	Hifiasm	436.10	677	31.68	4	58.32	99.22	99.21	99.75
	RAGA	417.32	251	31.93	0	59.60	99.22	99.34	100
Rice (HuaZhan)	Hifiasm	441.89	1038	31.90	2	54.62	99.08	99.27	100
	RAGA	422.01	442	31.96	2	55.61	99.06	99.22	100
Rice (J4155S)	Hifiasm	434.60	866	31.95	6	56.21	98.96	99.27	100
	RAGA	414.55	299	28.23	6	58.34	98.93	99.11	99.75
Rice (LK638S)	Hifiasm	419.83	552	30.95	8	57.86	98.92	99.10	99.50
	RAGA	404.85	158	32.49	3	61.41	98.90	99.22	99.50
Rice (XL628S)	Hifiasm	421.94	514	30.50	12	58.03	99.28	98.97	99.75
	RAGA	410.22	201	31.75	10	60.14	99.27	99.15	99.75
E. colona	Hifiasm	1314.72	9477	3.56	3648	59.31	98.92	95.16	100
	RAGA	1249.29	5874	6.06	2126	60.20	98.91	96.18	99.90
S. spontaneum	Hifiasm	3111.62	22 039	1.41	13 974	55.40	79.51	90.89	100
	RAGA	3062.67	17 119	1.93	11 384	56.18	79.51	92.22	99.95

**Figure 3:**
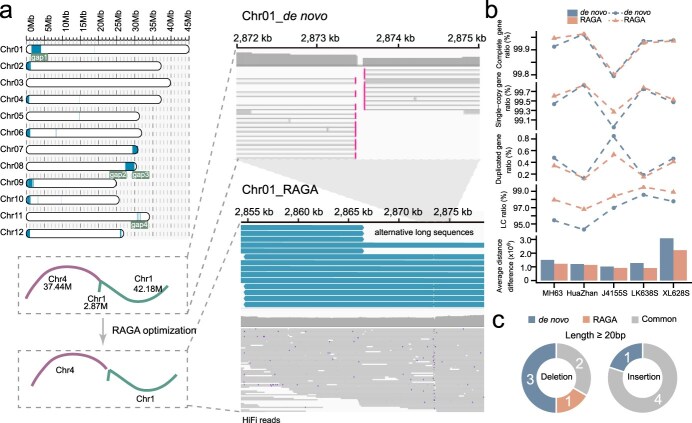
RAGA improves rice genome assembly. (a) Distribution of alternative long sequences generated by RAGA for MH63 using multiple reference genomes. The dashed box highlights the correction of a cross-chromosomal misassembly (Chr1 and Chr4) (left panel). Example of gap closure at gap_1 on chromosome 1 (2.87-Mb coordinate) achieved by RAGA-generated alternative long sequences (right panel). (b) Line and bar charts represent the differences between *de novo* and RAGA-optimized assemblies in the ratio of complete genes, single-copy complete genes, duplicated complete genes, proportion of the largest categories (LC), and average distance difference. (c) Structural variant comparison against the MH63 T2T reference genome, quantifying insertions and deletions (≥20 bp). The pie chart illustrates *de novo* assembly-specific variants (labeled as *de novo*), RAGA-optimized-specific variants (labeled as RAGA), and shared discrepancies (labeled as Common).

In k-mer evaluation with the MH63 T2T genome as a reference, the RAGA-optimized assembly performed better than *de novo* assembly by slightly increasing the single-copy completeness rate and average proportion of the largest category (Fig. 3 and Table S5). These enhancements indicate that the application of RAGA has improved the completeness and continuity of the assembly. Furthermore, *de novo* assembly exhibited a reduction in duplication completeness rate and a decrease in the average distance difference, suggesting that RAGA can increase the assembly accuracy and reduce redundancies (Fig. 3b and Table S5).

Our investigation of MH63 gap regions revealed that RAGA effectively addresses assembly challenges in repetitive sequences and complex genomic regions. The four gaps in the original MH63 *de novo* assembly (Fig. 3a) were characterized by high repeat content and ambiguous read mappings (Fig. S6). Gap-1 on chromosome 1 was caused by an incorrect fusion of reads from chromosomes 1 and 4 in *de novo* assembly (Fig. 3a). Similarly, the other three gaps on chromosomes 8 and 11 were caused by contigs that failed to successfully span complex assembly regions during the contig connection process (Fig. S5). By leveraging multiple T2T-level rice reference genomes, RAGA generated alternative long sequences that can effectively cross the gap regions on chromosomes 1 and 8, and produced crucial alternative long sequences near the gap-4 on chromosome 11. RAGA successfully resolved these issues by generating bridging sequences using multiple T2T rice references, resulting in continuous HiFi read coverage across previously gapped regions (Fig. S5), confirming its reliability for gap closure.

Comparative collinearity analysis of MH63 and NIP T2T genomes revealed a structural inversion spanning ~5 Mb on chromosome 6 (Fig. S6). To assess whether RAGA introduces structural variations into the assembly when there are significant structural differences between the reference genome and the target assembly, we disrupted the start and end points of the inversion on chromosome 6 of the MH63 T2T genome, thereby creating two gaps (Fig. S7). Subsequently, we applied RAGA to optimize the disrupted genome using the NIP T2T genome as the reference. Through collinear analysis between the RAGA-optimized assembly and the original MH63 T2T genome, we found that they were completely consistent on chromosome 6 (Fig. S8). RAGA generated alternative long sequences adjacent to the gaps without introducing errors (Fig. S9).

We further assessed RAGA’s performance across four additional rice varieties (HuaZhan, J4155S, LK638S, and XL628S) using NIP T2T genome as the reference, which resulted in reduced contig numbers and improved QV scores for all cultivars (Table 1). In the assembly assessment based on k-mers, RAGA increased the ‘proportion of the largest category’ and reduced the ‘average distance difference’ for all assemblies (Fig. 3b, Table S5). Through collinear analysis of both *de novo* assemblies and RAGA-optimized assemblies with the reference genome, it was found that the two assembly methods exhibited near-complete collinearity with the reference genome (Fig. S10), indicating that RAGA did not introduce structural variations.

To further rigorously evaluate RAGA’s performance, we conducted simulation studies using PBSIM3 [27] to generate 50× coverage of synthetic HiFi reads from the MH63 T2T genome. This approach addresses the inherent challenges in assessing assembly tools with real sequencing data, where true error profile remain unknown [27]. Employing these simulated reads along with T2T references (TIP, ZS97, HuaZhan, J4155S, LK638S, and XL628S), we produced both *de novo* and RAGA-optimized assemblies. Comparative analysis revealed three key findings: (i) Collinearity assessment demonstrated superior alignment of RAGA-optimized assemblies with the MH63 T2T reference, particularly in regions corresponding to gaps in *de novo* assemblies (Fig. S11), confirming RAGA’s precision in gap resolution. (ii) Variant analysis showed that while both assembly methods produced minor deviations from the reference, RAGA-optimized assemblies contained significantly fewer large indels (>20 bp) than *de novo* assemblies (Fig. 3c), highlighting RAGA’s ability to minimize structural variations. (iii) Read composition analysis verified that all final contigs exclusively comprised original HiFi reads, with no incorporation of RAGA-generated alternative sequences (Table S6), ensuring the biological fidelity of optimized assemblies. These simulation-based results provide robust validation of RAGA’s assembly improvement capabilities while maintaining genomic integrity, addressing fundamental challenges in long-read assembly evaluation.

### The performance of RAGA with phylogenetic species as reference genomes

Even without a high-quality reference genome from the same species, RAGA can enhance the genome assembly quality of non-model organisms by leveraging phylogenetically related species as reference genomes. RAGA can identify conserved syntenic regions across these species and improve the assembly quality of the target genome. This capability was validated through applications to two challenging polyploid systems: the allohexaploid *Echinochloa colona* [25] and autotetraploid *Saccharum spontaneum* [26]. For *E. colona* optimization, we employed multiple rice T2T genomes (NIP, ZS97, MH63, HuaZhan, J4155S, LK638S, and XL628S) as references, while using *Sacharrum rufipilum* [27] as the reference for *S. spontaneum*.

RAGA-optimized assemblies demonstrated substantial enhancements in genome continuity for both *E. colona* and *S. spontaneum*. Specifically, *E. colona* assembly achieved a ~2.50-Mb increase in contig N50 length along with 1522 fewer gaps. For *S. spontaneum* assembly, the contig N50 length increased from 1.41 to 1.93 Mb, with a reduction of 2590 gaps. Concurrently, genome assembly accuracy showed marked improvement in both species. The QV of *E. colona* assembly was increased from 59.31 to 60.20, and the R-AQI was improved from 95.16 to 96.18. For the *S. spontaneum*, the QV was improved from 55.40 to 56.18, and the R-AQI was improved from 90.89 to 92.22. Notably, read composition analysis confirmed that all optimized contigs exclusively contained original HiFi reads, with no incorporation of RAGA-generated sequences (Table S6), ensuring reference-derived artifacts were avoided. Other quality metrics remained comparable between RAGA-optimized and *de novo* assemblies (Table 1).

These findings establish RAGA as a robust solution for non-model organism genome assembly, effectively leveraging phylogenetic conservation while maintaining assembly purity. Therefore, even in the absence of a high-quality reference genome from the same species, we recommend using RAGA alongside high-quality genomes from phylogenetically related species to achieve optimized assembly.

### Optimization effects of RAGA on published genome assemblies with different heterozygosity

**Figure 4:**
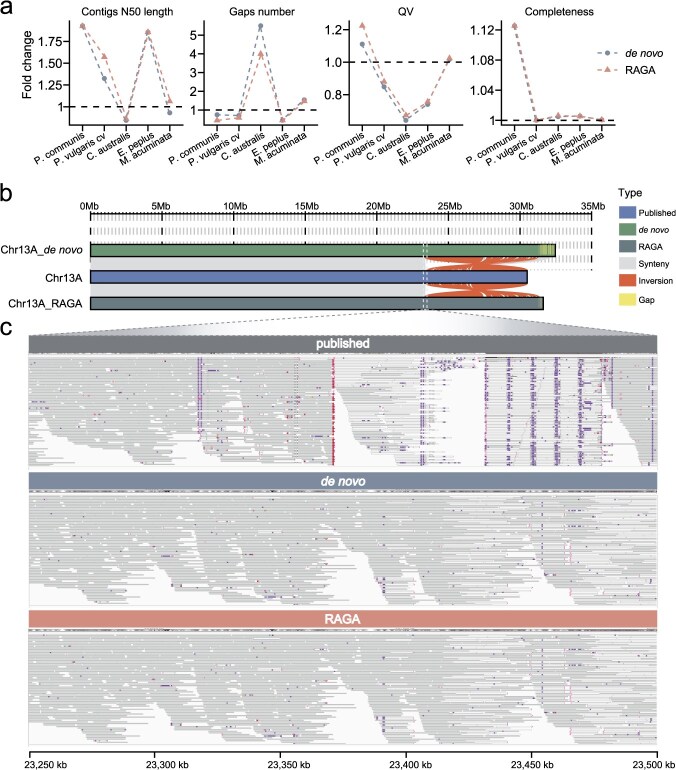
Quality comparison of RAGA-optimized assembly, *de novo* assembly and the published genome. (a) Line charts depicting the fold changes in assembly evaluation metrics for *P. communis*, *P. vulgaris cv*, *C. australis*, *E. peplus*, and *M. acuminata*, comparing *de novo* assembly (labeled as *de novo*) with RAGA-optimized assembly (labeled as RAGA) relative to the published genome. Assembly metrics include contig N50 length, gaps number, QV, and completeness. (b) Visualization of collinearity on Chr13A for the *de novo* assembly, published genome, and RAGA-optimized assembly of *P. communis*. (c) Alignment map of PacBio HiFi reads near the inversion initiation site on Chr13A of *P. communis* across different assemblies.

We further evaluated the performance of RAGA in enhancing genome assemblies across diverse organisms, ranging from low-heterozygosity diploids to complex polyploids. Comprehensive evaluations revealed that RAGA-optimized assemblies demonstrated consistent improvements in both assembly continuity (manifested as reduced contig counts and increased contig N50 length) and accuracy (quantified by higher QV scores) when benchmarked against *de novo* assemblies (Fig. 4a, Table S7). In addition, RAGA-optimized assembly has improved the quality of highly heterozygous *Pyrus communis* [28] compared with the published assembly, and enhanced the contig continuity of low-heterozygous genomes *Phaseolus vulgaris* cv. [29] and *Euphorbia peplus* [30] (Fig. 4, Table S7). In the case of the autotriploid *Musa acuminata* genome [31], the RAGA-optimized assembly successfully improved the base accuracy (Fig. 4a, Table S7).

In low-heterozygosity species, the RAGA-optimized assembly improved the continuity of the published genomes *P. vulgaris* cv. [29] and *E. peplus* [30], while reducing gap frequency. Nevertheless, the absence of a polishing step resulted in lower assembly accuracy compared to the existing published genomes. For *P. vulgaris* cv. genome, the RAGA-optimized assembly reduced the number of contigs by 589 and 903, and decreased the gap number by 4 and 14, therefore elevated contig N50 values to 4.85 and 11.29 Mb compared with the *de novo* assembly and the published genome [29], respectively. However, the QV metric was equivalent to the *de novo* assembly and slightly lower than that of the published genome, indicating that the *de novo* assembly still requires a polishing step. Nonetheless, RAGA effectively enhanced the continuity of the *P. vulgaris* cv. assembly (Fig. 4a, Table S7). In the case of *E. peplus* [30], RAGA-optimized assembly reduced the number of contigs, and the other features were largely comparable to those of the published and *de novo* assemblies (Fig. 4, Table S6). In the case of *Citrus australis* [32], the RAGA-optimized assembly demonstrated improved quality compared to the *de novo* assembly by reducing the number of gaps by three. However, when compared to the published *C. australis* genome, which contains only two gaps (representing a nearly complete genome assembly), the RAGA-optimized assembly did not achieve further quality enhancement.

For the highly heterozygous diploid genome *P. communis* [28], our analysis showed that the RAGA-optimized and *de novo* assemblies both outperformed the published genome in all quality metrics. Compared with *de novo* assembly, the RAGA-optimized assembly decreased the number of gaps by 42 (Fig. 4a, Table S7). Collinearity analysis revealed a ~7-Mb inversion within 23-Mb region on chromosome 13A in both the *de novo* assembly and RAGA-optimized assembly compared to the published genome (Fig. 4b). Through alignment of PacBio HiFi reads, it was found that reads in this region could continuously cover both the *de novo* assembly and RAGA-optimized assembly, whereas the published genome exhibited distinct breakpoints in PacBio HiFi read coverage (Fig. 4c). This indicates that the assemblies have corrected the inversion errors present in the published genome. Moreover, the RAGA-optimized assembly reduced the number of gaps in Chr13A from 23 (in the *de novo* assembly) to 3, suggesting that RAGA-optimized assembly can further enhance assembly continuity compared with the *de novo* assembly (Fig. 4b). This result confirms the efficacy of RAGA in enhancing the quality of pear diploid genome assembly, making it suitable for assembling highly heterozygous diploid genomes.

In the case of autotriploid *M. acuminata* genome [31], RAGA-optimized assembly demonstrated improved continuity compared to the *de novo* assembly. Specifically, it reduced the number of contigs, increased contig N50, and decreased the number of gaps (Fig. 4a and b, Table S7). However, when compared with published genomes, the RAGA-optimized assembly failed to reduce the number of gaps. Collectively, these findings indicate that RAGA is a valuable tool for generating more contiguous and higher quality genomes.

## Discussion

### Performance under HiFi & ONT hybrid assembly

For the assembly of complex genomes, HiFi and ONT sequencing technologies are frequently used together. To evaluate whether RAGA enhances assembly quality in hybrid sequencing scenarios, we used the soybean variety ZH13 [33], kiwifruit cultivar ‘Hongyang’ [34], and human genome HG002 [35] to assess the impact of RAGA on assembly quality by employing a hybrid approach with both HiFi and ONT reads.

Using the T2T genomes of soybean varieties Williams 82 [36] and Jack [37] as references, we optimized the assembly of ZH13 using RAGA. The results indicate that when only HiFi reads are assembled, the RAGA-optimized assembly improves assembly quality compared to *de novo* assembly. However, when compared with the hybrid assembly of HiFi and ONT reads, RAGA fails to further enhance the assembly quality (Table S8). Additionally, statistical analysis of the read source of ZH13 contigs revealed that during hybrid assembly, only one alternative long sequence generated by RAGA was introduced into the contigs (Table S6). This RAGA alternative long sequence is covered by uniform and continuous HiFi reads (Fig. S12) and did not introduce assembly errors from the reference. For kiwifruit cultivar ‘Hongyang’ [34], the RAGA-optimized assembly did not achieve quality improvements compared to the hybrid assembly of HiFi and ONT reads. Similarly, when using CHM13 [38] as the reference for the *de novo* hybrid assembly of HG002 [35], no enhancement in assembly quality was observed in the RAGA-optimized assembly (Table S9). Consequently, for samples sequenced with both HiFi and ONT reads, we do not recommend employing RAGA for assembly optimization, as it only increases computational resource but cannot significantly improve the assembly quality.

### The significance and limitations of RAGA

In the current era of pan-genomics, the demand for multiple, fine-grained assemblies at the T2T level has significantly increased [1]. However, traditional approaches for fine-grained genome assembly are confronted with the challenges of high sequencing costs [10] and substantial computing resources [39]. To overcome these limitations, we present the RAGA tool, which can integrate T2T reference genomes into the *de novo* assembly of target genomes.

The objective of RAGA is to introduce a reference genome during *de novo* assembly to enhance the overall assembly quality while avoiding biases introduced by the reference genome. Unlike other tools that only utilize the reference genome during scaffolding stage (such as RagTag [15] and quarTeT [16]), RAGA employs a unique hybrid assembly approach that integrates the reference genome into the assembly process. This methodology improves both sequence accuracy and continuity in genomic assemblies. Moreover, this approach not only boosts the overall quality of the assembly but also simplifies and lightens the workload for subsequent assembly procedures. Recent study on the pan-genome of 22 *Solanum* species (based on PacBio HiFi reads) revealed the critical role of homologous gene diversification in trait variation [40]. Therefore, carrying out pan-genome studies on horticulturally significant crops is crucial. The use of RAGA can significantly improve the quality of population-scale genome assemblies, thus facilitating pan-genomic research in horticultural crops.

While RAGA achieves substantial advancements across multiple metrics, the methodology also has limitations. Firstly, the phylogenetic relationship between the reference and the target genome can affect the performance of RAGA. When applied to rice MH63 assembly using multiple rice T2T genomes as references, RAGA-optimized workflows can achieve gap-free genomes. However, in the RAGA-optimized assembly of *E. peplus* using cassava XX048 T2T genome as a reference, the improvement in assembly quality by RAGA is minimal. This is because when the phylogenetic relationship between the reference and the target genome is distant, there are only a few high-quality alignment blocks between them. Under such conditions, RAGA can only generate a small number of shorter alternative long sequences, limiting the improvement in assembly quality. Additionally, for complex polyploid assemblies, although RAGA provides measurable improvements over conventional *de novo* approaches, its assistance in the overall polyploid assembly process remains constrained. Furthermore, compared to *de novo* assembly, RAGA-optimized assembly increases the runtime of the assembly process (Table S10). Lastly, regarding technical implementation, RAGA’s localized hybrid assembly module is based on hifiasm (UL) [41]. However, the study did not explore the optimal configuration parameters for hifiasm (UL) due to the significant workload involved and the possibility that the optimal parameters may vary depending on the species.

Considering the aforementioned limitations of RAGA, we recommend that researchers conduct comprehensive quality assessments of *de novo* assemblies prior to implementing RAGA. If the *de novo* assembly already approaches genome completeness, applying RAGA may not significantly enhance assembly quality and could instead lead to unnecessary computational resources consumption. Furthermore, when utilizing phylogenetically distant species as references, the phylogenetic distance between reference and target genomes must be carefully evaluated. For cross-genus species pairs where reference and target genomes belong to distinct genera, RAGA’s optimization effect may become unstable.

RAGA generates alternative long sequences by leveraging reference genomes and PacBio HiFi reads, thereby replacing ONT reads. Consequently, in hybrid assemblies incorporating both PacBio HiFi and ONT sequencing data, RAGA cannot further improve assembly quality. We attempted to develop an next-generation sequencing (NGS)-compatible version of RAGA (RAGA-NGS), but yeast genome assembly tests revealed that RAGA-NGS optimized assemblies still exhibited inferior quality compared to PacBio HiFi-based assemblies. Additionally, compared with MetaCompass [42], RAGA is unsuitable for metagenomic assembly. Given the increasing availability of T2T reference genomes and the cost-effectiveness of NGS technologies, we plan to continue optimizing the RAGA-NGS functionality.

The recent publication of a T2T assembly for hexaploid bread wheat [43] underscores the persistent complexity of polyploid genome assembly. In this study, we evaluated RAGA’s optimization effects on PacBio HiFi-based *de novo* assemblies across several polyploid species, including autotriploid *M. acuminata*, autotetraploid *S. spontaneum*, and allohexaploid *E. colona*. Results demonstrated that RAGA’s capacity to enhance polyploid assembly quality remains limited. This limitation partially stems from the frequent presence of multiple haploid genomes or subgenomes in polyploids, which can introduce ambiguity during the search for high-quality alignment blocks and ultimately compromise the generation of effective alternative long sequences. Polyploidy is prevalent among key horticultural crops, with cultivated strawberry (*Fragaria* × *ananassa*) representing a classic allooctoploid system comprising four differentiated subgenomes (ABCD) [44]. Future efforts will focus on implementing haplotype phasing for polyploid genomic reads prior to RAGA processing, aiming to enhance RAGA’s optimization performance in polyploid assemblies.

## Conclusions

In summary, the quality of assembly at the population scale is crucial for pangenome analysis. In response to the current situation in pangenome research, where it is difficult to perform PacBio HiFi reads and ONT ultralong reads sequencing on all samples simultaneously, RAGA can introduce a reference genome into *de novo* assembly and generate simulated ONT ultralong reads based on the reference genome. Based on extensive testing, we demonstrate that the use of RAGA can improve the quality of assembly and simplify the assembly process. Ultimately, the application of RAGA in population-scale genome assembly will contribute to the pangenome studies, which can be freely available at https://github.com/wzxie/RAGA.

## Methods

### Workflow of RAGA

RAGA aims to generate long sequences that can be used as ONT reads for the target assembly involving PacBio HiFi reads, based on closely related high-quality reference genome. The generated long sequences can be used as ONT reads to participate in the *de novo* assembly of the target genome, thereby improving the quality of its genomic assembly.

The RAGA workflow consists of four steps: correcting reference, alignment, localized hybrid assembly, and filtering.

In the correcting reference step, when the reference genome and target genome belong to the same species, the ‘map-hifi’ parameter of minimap2 (v2.22-r1101) [45] is first used to align the HiFi reads with the target contigs assembly. Target haplotype HiFi reads are then filtered based on the alignment file. The reference genome is aligned with the target haplotype HiFi reads using ‘map-hifi’ parameter of minimap2 (v2.22-r1101), and the reference genome is polished based on the target haplotype HiFi reads using racon (v1.4.20) [46] according to the alignment file, resulting in a mixed genome containing both reference genome and target genome fragments. If the reference genome and target genome belong to phylogenetic species, the ‘map-hifi’ parameter of minimap2 (v2.22-r1101) is directly used to align the reference genome with the HiFi reads, and the reference genome is polished based on the HiFi reads using racon (v1.4.20) according to the alignment file, resulting in a mixed genome.

In the alignment step, when the reference and target genome belong to the same species, RagTag (v2.1.0) [15] is used to scaffoldold the contigs based on the mixed genome. Nucmer (4.0.0rc1) [47] is then used to perform collinear alignment between the mixed genome and target scaffolds. Delta-filter and show-coords [47] are used for filtering and format conversion, ultimately retaining high-quality alignment blocks near the gaps in the target scaffolds. When the reference and target genome belong to phylogenetic species, minimap2 (v2.22-r1101) [45] with the ‘map-hifi’ parameter is used to directly align the target HiFi reads with the mixed genome. All alignments are filtered to retain only those with a primary alignment tag of ‘tp:A:P’ and an accurate alignment length accounting for 99% of the total read length. Bedtools (v2.26.0) [48] is then used to merge each filtered alignment region and filter out shorter alignments.

In the localized hybrid assembly step, minimap2 (v2.22-r1101) with the ‘map-hifi’ parameter is used to align the target HiFi reads to the mixed genome. Based on the coordinates of high-quality alignment blocks in the mixed genome, target HiFi reads with overlap in alignment positions are extracted to obtain target HiFi reads for each alignment block. Simultaneously, corresponding mixed genome sequences are extracted as ONT ultralong sequences for each alignment block based on their coordinates in the mixed genome. Hifiasm (v0.19.9) [41] with default parameters is then used to perform localized hybrid assembly on the target HiFi reads and ONT ultralong sequences for each alignment block, completing the generation of original long sequences. We filter the localized hybrid assembly in subsequent steps for rigorousness.

In the filtering step, the original long sequences generated in the previous step are filtered. When the reference and target genome belong to the same species, minimap2 (v2.22-r1101) [45] with the ‘map-hifi’ parameter is first used to align the target HiFi reads within each alignment block to the corresponding localized hybrid assembly. Samtools (v1.3.1) [49] is used to statistically analyze the read coverage of each localized assembled sequence, filtering out sequences with coverage breakpoints. Next, minimap2 with the ‘asm5’ parameter is used again to align the filtered localized assembled sequences within the alignment block to the target contigs, further filtering out localized assembled sequences that are completely identical or significantly different from the target contigs. Finally, shorter localized assembled sequences are filtered out to complete the generation of final long sequences for RAGA. When the reference and target belong to phylogenetic species, minimap2 with the ‘map-hifi’ parameter aligns the target HiFi reads from each alignment block to their localized assembled sequences. Samtools (v1.3.1) [49] with default parameters is then used to statistically analyze the alignment results, filtering out localized assembled sequences with breakpoints in target HiFi reads coverage. Finally, shorter localized assembled sequences are filtered out to complete the generation of RAGA’s final long sequences.

### Filtering of sequencing reads

For the preprocessing of NGS reads, including both standard and HiC reads, we utilize fastq (v0.12.4) [50] to rigorously filter out artifacts such as PCR duplicates, low-quality sequence fragments at the 5′ and 3′ termini of the reads, as well as reads of overall poor quality. Specifically, this involves removing reads with low Phred scores or those containing ambiguous bases.

In the case of third-generation sequencing reads, we employ nanoqc (v0.9.4) and nanofilt (v2.8.0) [51] for preprocessing. These tools allow us to systematically discard reads with low-quality ends or those that fail to meet our stringent quality thresholds. This filtering process ensures that only high-quality reads are retained for subsequent bioinformatics analyses, thereby enhancing the accuracy and reliability.

### RAGA-optimized assembly for *A. thaliana* population

To evaluate the performance of RAGA tool across different population sizes, we collected PacBio HiFi reads data from 80 *A. thaliana* populations [17, 18] sourced from a pan-genome study. We performed *de novo* assembly on these 80 samples using the default parameters of hifiasm (v0.19.9) [41]. Subsequently, using three published T2T genomes of *A. thaliana* [20–22] as references, we generated long sequences for these 80 samples using the RAGA tool. These long sequences were then treated as ONT reads and mixed with their respective PacBio HiFi reads for hybrid assembly, which was conducted based on hifiasm (v0.19.9) [41].

### RAGA-optimized assembly for various genomes

To further validate the broad applicability of the RAGA tool, we conducted experiments on rice, soybean, *E. colona* [52], and *S. spontaneum* [53]. Initially, we gathered PacBio HiFi reads from the rice variety MH63 [24] and performed *de novo* assembly using hifiasm (v0.19.9) [41]. Meanwhile, selecting multiple rice T2T genomes (such as TIP [25], ZS97 [24], HuaZhan [26], *etc*.) as references, we generated alternative long sequences using RAGA and performed hybrid assembly by integrating original PacBio HiFi reads with hifiasm (v0.19.9) [41].

To evaluate the impact of structural variations on RAGA, the study used seqkit (v2.5.1) [54] to fragment the MH63 T2T genome and performed RAGA-optimized assembly using the TIP T2T genome as a reference.

Based on PacBio HiFi reads from HuaZhan, J4155S, LK638S, and XL628S, the study conducted *de novo* assembly using hifiasm (v0.19.9) [41]. Additionally, RAGA-optimized assembly was obtained using the RAGA process, with the TIP T2T genome serving as a reference.

Based on the MH63 T2T genome, ~50× simulated PacBio HiFi reads were generated using PBSIM3 [27]. Following this, *de novo* assembly was performed using hifiasm (v0.19.9) [41]. With the T2T genomes of TIP, ZS97, HuaZhan, J4155S, LK638S, and XL628S as references, RAGA-optimized assembly was obtained using RAGA.

To investigate whether RAGA conflicts with ONT reads, we collected PacBio HiFi reads and ONT reads from soybean ZH13 [33], performing both HiFi-only *de novo* assembly and HiFi & ONT hybrid assembly. Using T2T genomes of soybean varieties Williams 82 [36] and Jack [37] as references, we employed RAGA to generate alternative long sequences, which were then added to the *de novo* assembly data for reassembly based on hifiasm (v0.19.9) [41].

We collected PacBio HiFi reads and ONT reads from kiwifruit ‘Hongyang’ [34], performing both HiFi-only *de novo* assembly and HiFi & ONT hybrid assembly using hifiasm (v0.25.0) [41]. Using T2T genomes of kiwifruit varieties ‘DH’ as references [55], we employed RAGA to generate alternative long sequences, which were then added to the *de novo* assembly data for reassembly based on hifiasm (v0.25.0) [41].

We collected PacBio HiFi reads and ONT reads from humam ‘HG002’ [35], performing both HiFi-only *de novo* assembly and HiFi & ONT hybrid assembly using hifiasm (v0.25.0) [41]. Using T2T genomes of human ‘CHM13’ as references [38], we employed RAGA to generate alternative long sequences, which were then added to the *de novo* assembly data for reassembly based on hifiasm (v0.25.0) [41].

To evaluate the performance of RAGA when using phylogenetic species as reference genomes, the study collected PacBio HiFi reads from the allohexaploid *E. colona* [52] and the autotetraploid *S. spontaneum* [53]. *De novo* assembly of *E. colona* was performed using hifiasm (v0.19.8-r603) [41], while *de novo* assembly of *S. spontaneum* was performed using hifiasm (v0.19.9). The *de novo* assembly of *E. colona* was optimized using RAGA, with all published rice T2T genomes (including TIP, ZS97, MH63, HuaZhan, J4155S, LK638S, and XL628S) serving as reference genomes. Similarly, the *de novo* assembly of *S. spontaneum* was optimized using RAGA, with the *S. rufipilum* [56] genome as the reference.

### Quality improvement tests on published genomes

To assess the effectiveness of the RAGA tool in enhancing the quality of published genomes, we gathered published genomes and corresponding PacBio HiFi reads for *P. communis* [28], *P. vulgaris cv* [29], *C. australis* [32], *E. peplus* [30], and *M. acuminata* [31]. Firstly, we performed *de novo* assembly using hifiasm (v0.19.8-r603) [41]. Specifically, YunhongNO.1 [57] served as the reference genome for *P. communis*; soybeans Williams 82 [36], Jack [37], and ZH13 [33] were used as reference genomes for *P. vulgaris cv* [29]; T2T lemon [58] was the reference for *C. australis* [32]; cassava XX048 T2T genome [59] was the reference for *E. peplus*; and banana Cavendish T2T genome [60] was the reference for *M. acuminata* [31]. After running RAGA, we treated the long sequences outputted by RAGA as ONT reads and mixed them with the original PacBio HiFi reads for hybrid assembly, resulting in the RAGA-optimized assembly.

### Evaluation of assembly continuity

To assess the continuity of the assembly, we first employed the seqkit (v2.5.1) [54] tool and calculated N50 length, the number of contigs, and the average length, which provided fundamental data on assembly continuity. Subsequently, utilizing the ragtag (v2.1.0) [15] software and published reference genomes, we scaffolded the initially assembled contigs and counted the number of gaps generated after scaffolding, serving as a crucial indicator for evaluating assembly continuity.

### Evaluation of assembly completeness and accuracy

We adopted multiple methods for a comprehensive evaluation of assembly completeness and accuracy. Initially, we used the BUSCO tool (v5.5.0) [23] to assess completeness based on single-copy orthologous genes. Secondly, we conducted a k-mer analysis with Merqury (v1.3) [61], quantifying the assembly quality by calculating base accuracy and assembly completeness.

To further validate the assembly’s accuracy, we used the CRAQ (v1.0.9) [62] to predict structural errors. Moreover, we implemented collinear visualization of the assembly with the Genomesyn tool (v1.2.7) [63], providing us with an intuitive way to understand the assembly results. We used Minimap2 (v2.22-r1101) [45] to align HiFi reads with multiple assemblies and visualized specific regions using the Integrative Genomics Viewer (IGV) (v2.17.4) [64]. For testing of T2T assembly implemented in the published article, we conducted k-mer-based assembly evaluation using benchmark [65] based on the T2T reference genome.

For the *A. thaliana* population, mummer4 [47] was used to align different assemblies with reference genomes (including Col-0, Col-CEN, and Col-PEK), and SNPs variations were counted. Minimap2 (v2.22-r1101) [45] was used to align PacBio HiFi reads from different samples with the reference genomes (including Col-0, Col-CEN, and Col-PEK), and SNP variations were counted using bcftools (v1.20) [49]. Use pseudohaploid (https://github.com/schatzlab/pseudohaploid) to remove redundancy from assemblies and calculate the proportion of redundant sequences. Use BLAST [66] to align the CEN180 sequences against both *de novo* assemblies and RAGA-optimized assemblies, and perform statistical analysis.

For the simulated HiFi test of rice MH63, we aligned both *de novo* assembly and RAGA-optimized assembly to MH63 T2T reference genome using minimap2 (v2.22-r1101) [45]. The syri tool (v1.6.3) [67] was used to process the alignment results, obtaining detailed variation information for both assemblies compared to MH63 T2T reference genome.

## Supplementary Material

Web_Material_uhaf207

## Data Availability

The entire code and usage instructions for RAGA can be obtained at (https://github.com/wzxie/RAGA). All assemblies and raw reads used in this study are available in public databases. The 32 *A. thaliana* genome assemblies can be accessed at (https://figshare.com/articles/dataset/32_ecotypes_Arabidopsis_thaliana_genomes_gene_annotation_pan-TE_library_graph_pan-genome_gene_family_and_gene_presence_absence_matrices_files_/21673895), and the corresponding raw reads can be found at PRJCA012695 in the National Genomics Data Center (NGDC) [17, 68]. The PacBio HiFi reads for the additional 48 *A. thaliana* samples can be accessed in EMBL-ENA under the accession number PRJEB62038 [18]. The genome assemblies for these samples can be accessed in NCBI under the accession number PRJNA1033522 [18]. The published T2T *A. thaliana* genome is available at GWHBDNP00000000.1 in NGDC, (https://github.com/schatzlab/Col-CEN), and PRJCA007112 in NGDC [20–22]. The assembly and raw reads for rice MH63 can be accessed at CP054676-CP054688, SRX6957825, SRX6908794, SRX6716809, and SRR13285939 in the National Center for Biotechnology Information (NCBI) [24]. The published T2T genome for rice is available at CP056052-CP056064, CP054676-CP054688 in NCBI, and PRJCA018610 and PRJCA008812 in NGDC [24, 25]. The PacBio HiFi reads for Xiangling628S (XL628S), Longke638S (LK638S), and Jing4155S (J4155S) can be obtained from PRJCA008812 in the NGDC [26, 33]. The raw reads and assembly for soybean ZH13 can be found at PRJCA015269 and GWHBWDJ00000000.1 in NGDC [33]. The assemblies for soybean Williams 82 and Jack are available at PRJNA975879 and PRJNA701655 in NCBI [36, 37]. The T2T genome for *S. rufipilum* can be found at PRJCA014818 in NGDC [56]. The raw reads for *S. spontaneum* genome are available at PRJNA721787 in NCBI [53] . The raw reads for *E. colona* genome are available at PRJCA003883 in NGDC [52]. The assembly and raw reads for *P. communis* can be accessed at PRJNA992953 in NCBI [28]. The assembly and raw reads for *P. vulgaris cv* are available at PRJNA931244 in NCBI [29]. The raw reads for *C. australis* can be found at PRJNA910964 in NCBI, and the assembly is available at PRJCA013889 [32]. The raw reads and assembly for *E. peplus* [30] are available at PRJNA837952 in NCBI. The assembly and raw reads for *M. acuminata* [31] can be accessed at PRJNA1017453 in NCBI. The T2T genome for YunhongNO.1 can be found at (http://pyrusgdb.sdau.edu.cn/) [57]. The T2T lemon genome is available at GWHCBFQ00000000.1 in the CNCB Genome Warehouse [58]. The cassava XX048 T2T genome can be accessed at PRJCA016162 in NGDC [59], and the banana Cavendish T2T genome is available at PRJNA957115 in NCBI [60]. The raw reads and assembly for kiwifruit ‘Hongyang’ [34] can be obtained from the NCBI Sequence Read Archive (SRA) under the accession number PRJNA869178. The genome of kiwifruit ‘Donghong’ is available at the NGDC (National Genomics Data Center) with the Bioproject ID PRJCA014123. The genome and raw reads for human ‘HG002’ can be accessed via https://github.com/marbl/hg002, and the genome of human ‘CHM13’ is available at https://github.com/marbl/CHM13.
