# Supplementary material for: RAGA: a reference-assisted genome assembly tool for efficient population-scale assembly
Source: Hortic Res. 2025 Aug 11;12(11):uhaf207. doi: 10.1093/hr/uhaf207 (PMC12577851; doi:10.1093/hr/uhaf207)
Supplement: Web_Material_uhaf207 [file web_material_uhaf207.zip › Additional_file_1-Figure_S1-S12.pdf]

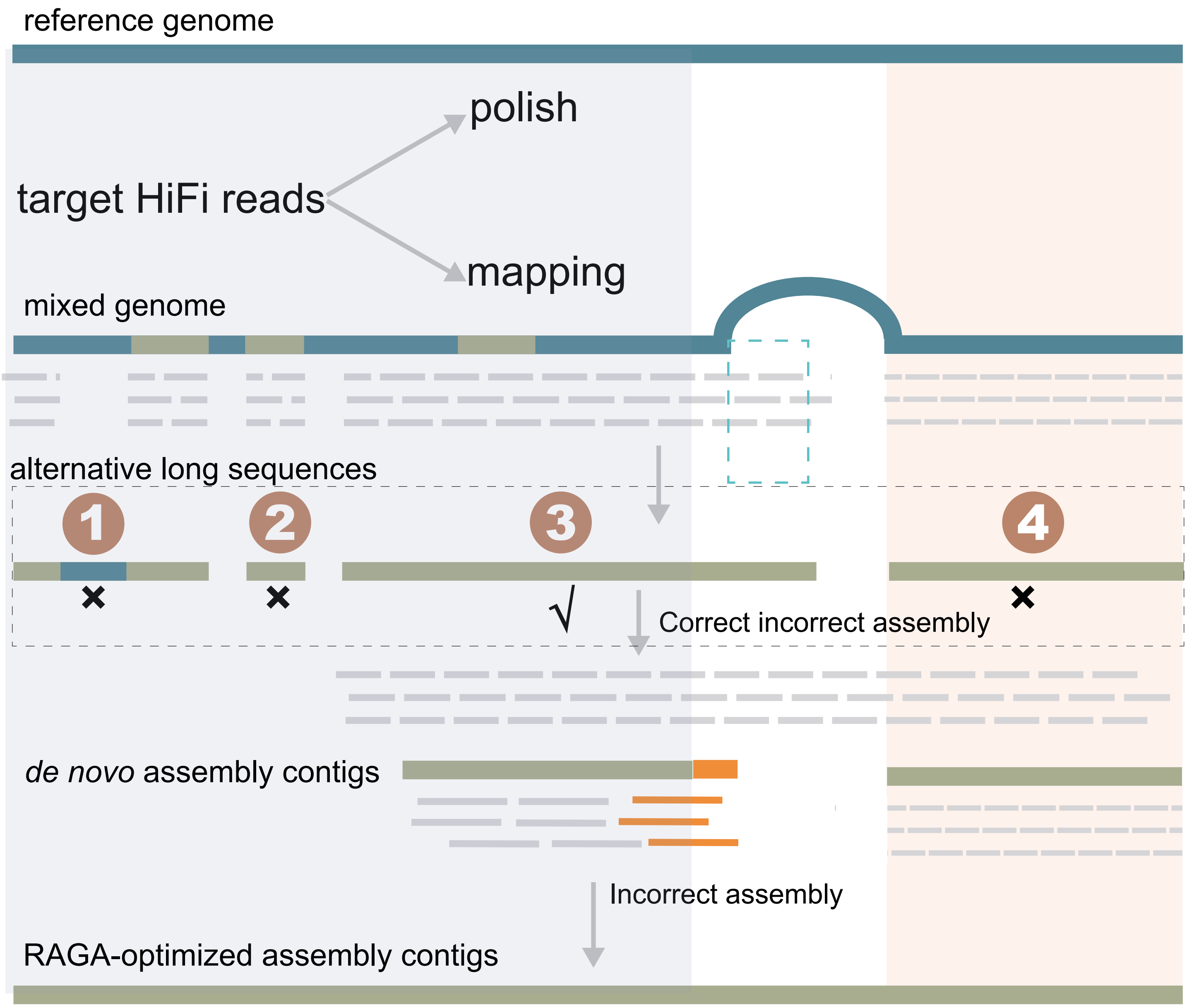

Additional file 1: Figure 1. Schematic diagram of RAGA assisting in solving assembly errors. RAGA generates long sequences that extend into the assembly error regions. These long sequences effectively resolve previous sequence connection issues during the *de novo* assembly process of the target genome, thereby significantly improving the quality of the assembly.

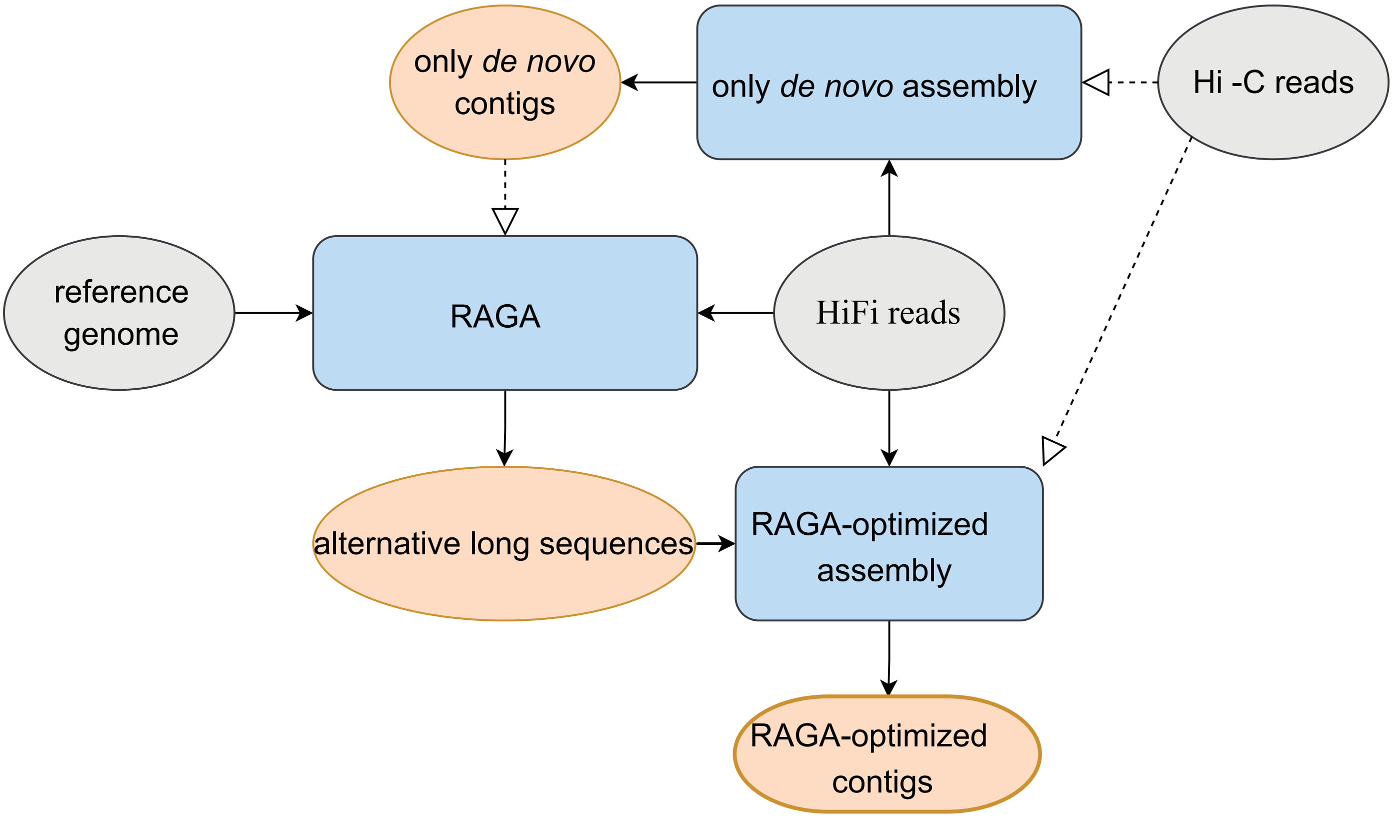

Additional file 1: Figure 2. The flowchart illustrates the method of using RAGA to upgrade *de novo* assembly to a RAGA-optimized assembly.

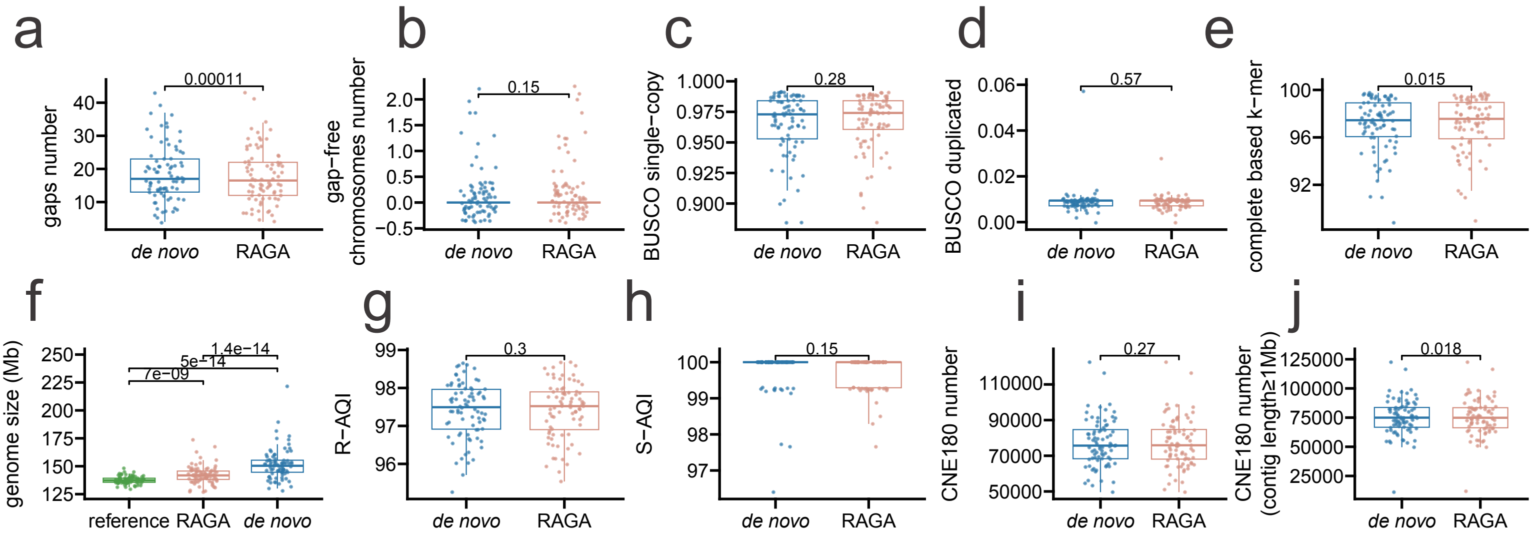

Additional file 1: Figure 3. Statistics of assembly indicators: (a) total number of gaps, **(b)** the number of gap-free chromosomes, **(c)** and **(d)** BUSCO assessments: single-copy orthologues and duplicated, **(e)** completeness evaluation based k-mer, **(f)** assembly size, **(g)** regional assembly quality index (R-AQI), **(h)** structural assembly quality index (S-AQI), **(i)** number of centromeric CNE180 sequence, and **(j)** number of centromeric CNE180 sequence (contig length ≥ 1Mb) the *Arabidopsis thaliana* population, including only *de novo* assembly and RAGA-optimized assembly. The median values, along with the interquartile range (IQR), are displayed within the boxes, while the whiskers indicate the data range spanning from the 10th to 90th percentiles. *P*-values obtained from the Wilcoxon test are also presented.

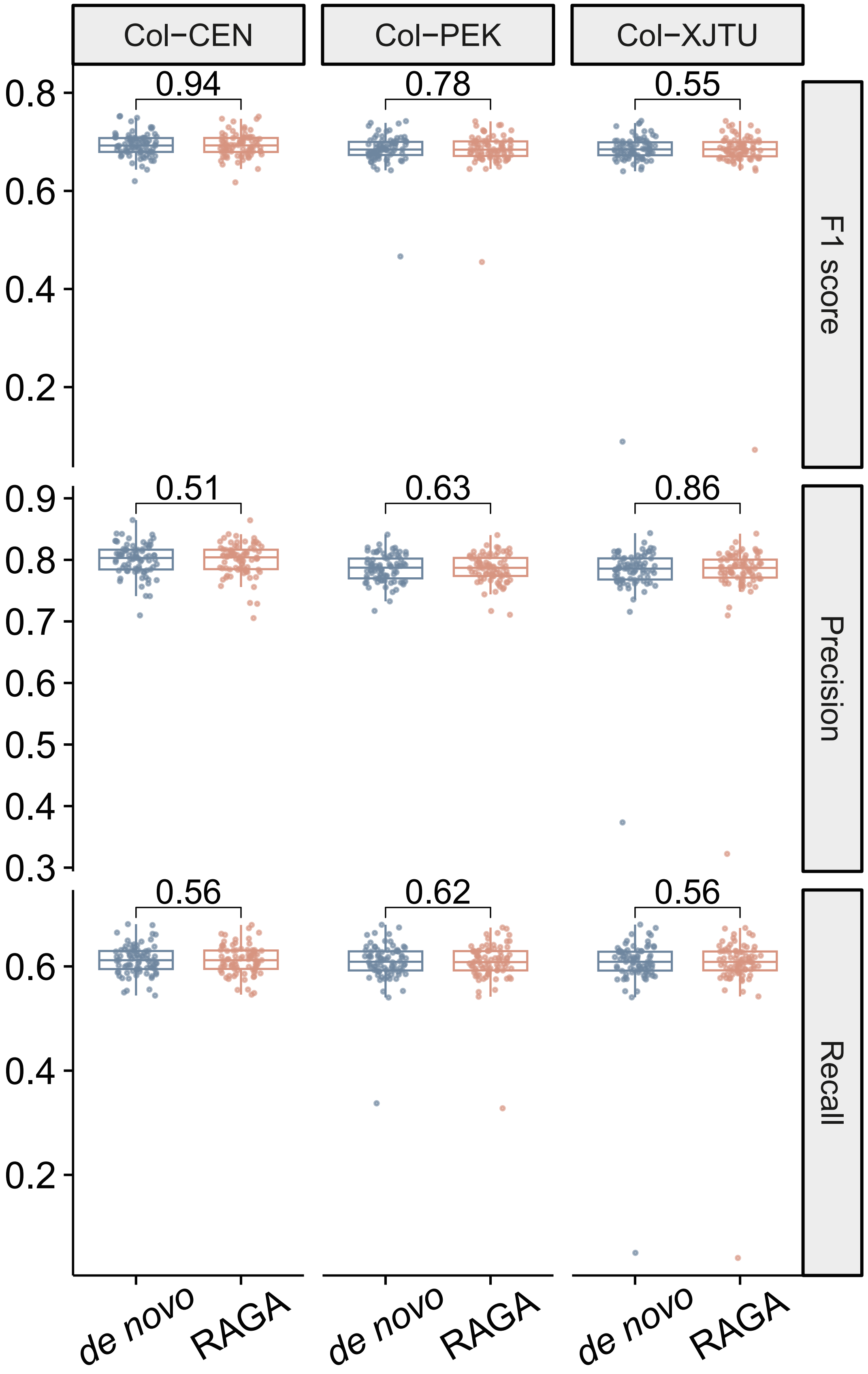

Additional file 1: Figure 4. Using three *A. thaliana* genomes (Col−CEN, Col−PEK, and Col − XJTU) as references, the differences in SNP calling between RAGA-optimized and *de novo* assemblies were evaluated using F1 score, Precision, and Recall. The median values, along with the interquartile range (IQR), are displayed within the boxes, while the whiskers indicate the data range spanning from the 10th to 90th percentiles. *P*-values obtained from the Wilcoxon test are also presented.

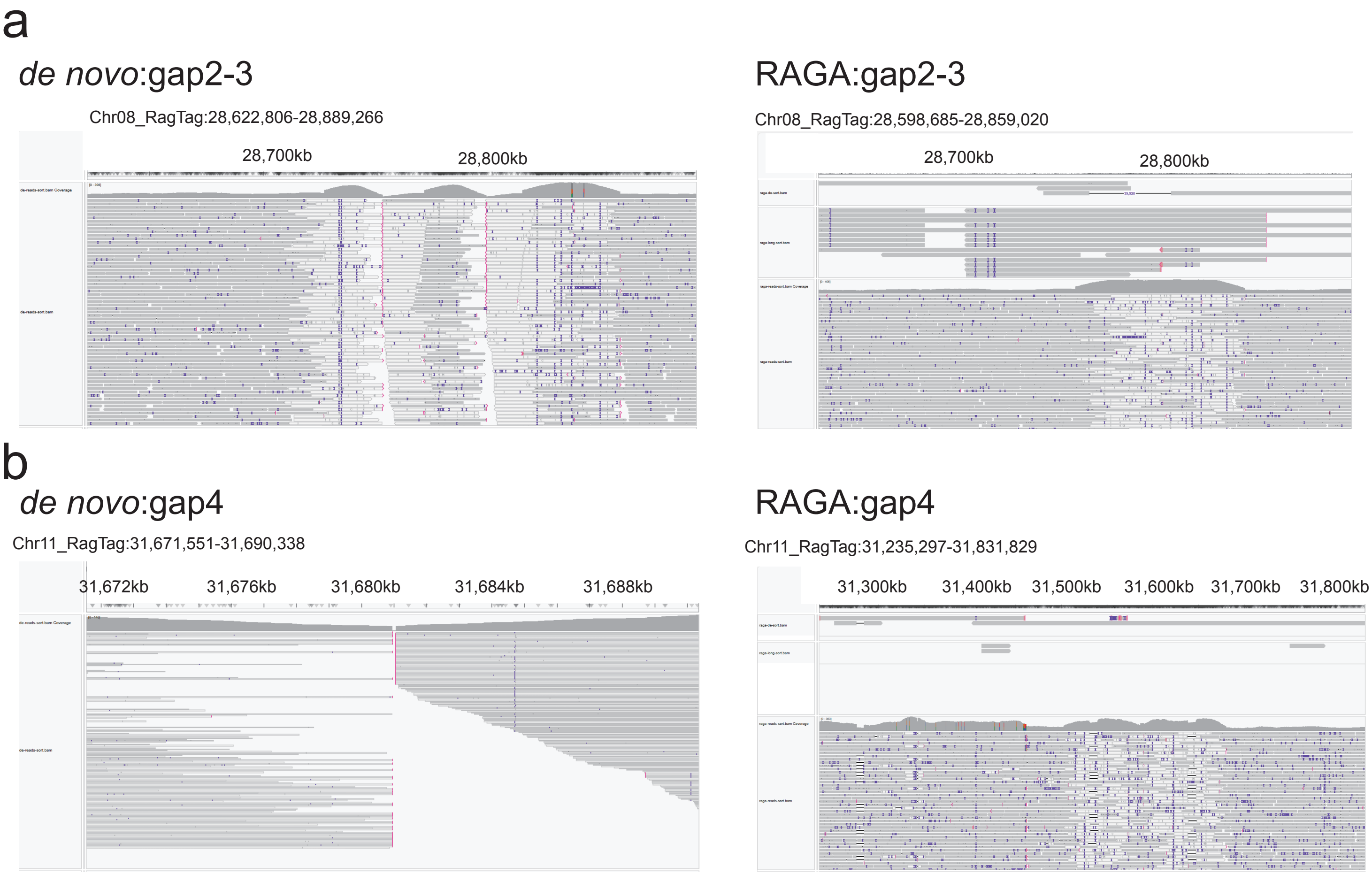

Additional file 1: Figure 5. Reads re-mapping of the gap regions on chromosomes (a) 8 and **(b)** 11 in the MH63 *de novo* assembly, and the reads re-mapping to RAGA-optimized assembly.

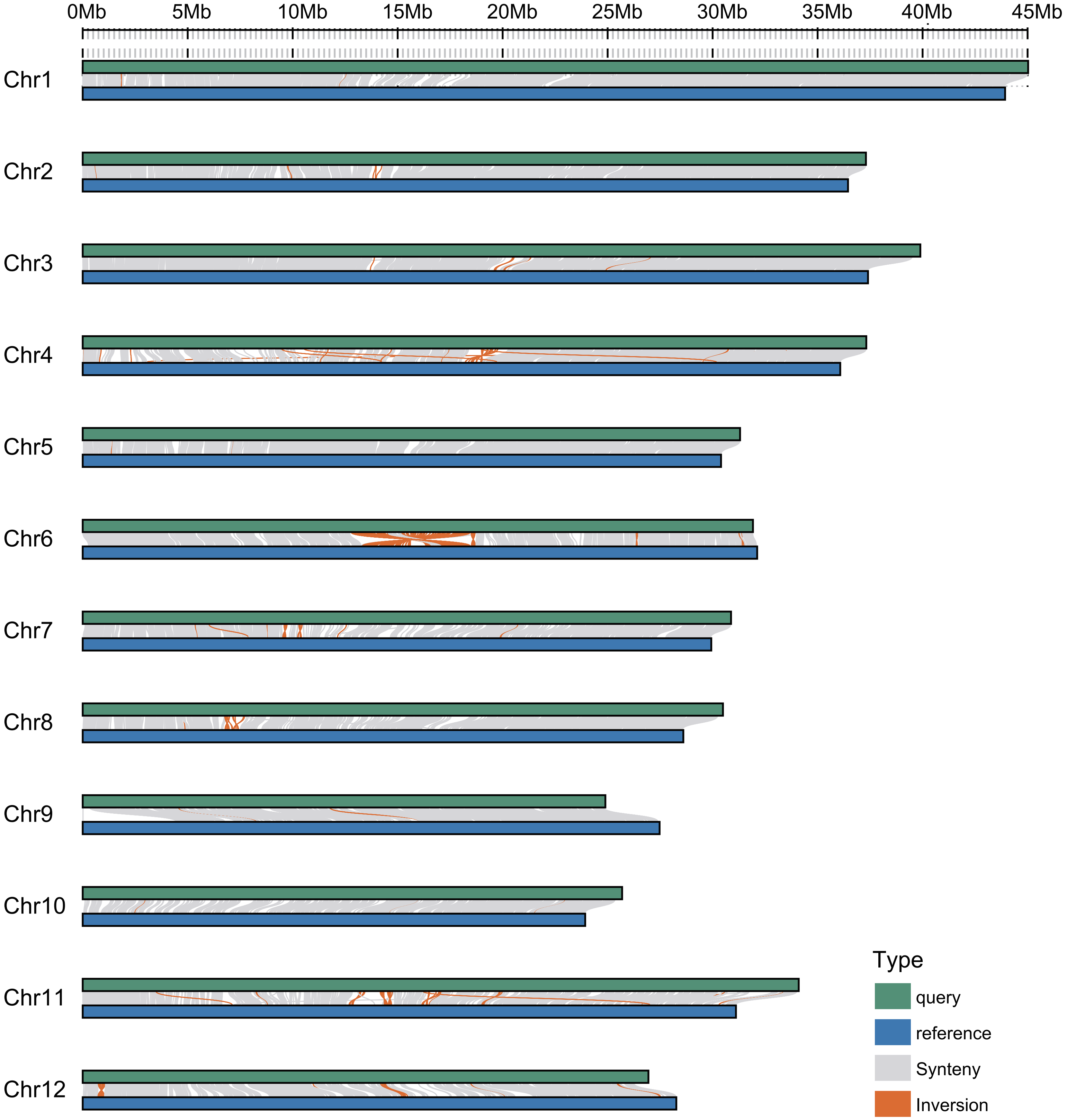

Additional file 1: Figure 6. Collinear analysis between the MH63 T2T genome and the NIP T2T genome revealed the presence of an inversion spanning approximately 5 Mb on chromosome 6.

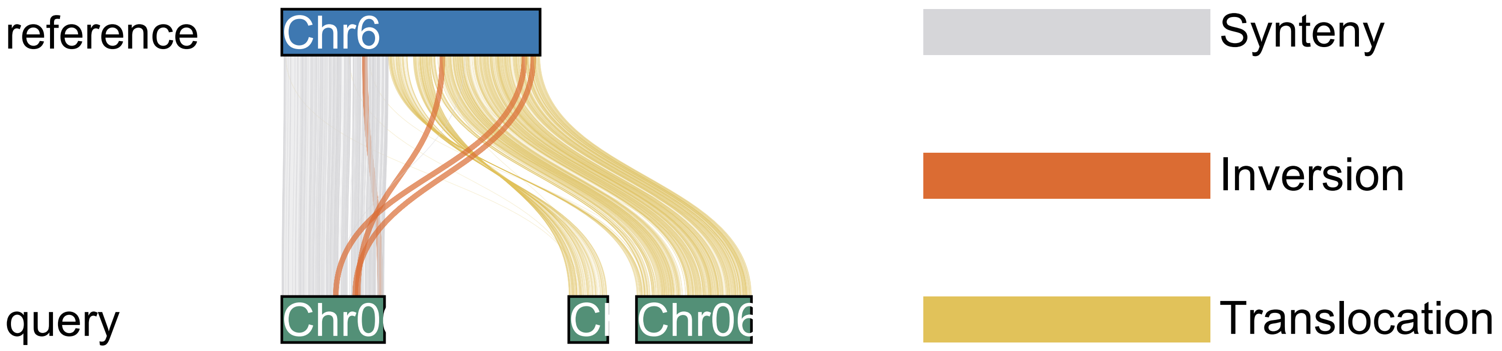

Additional file 1: Figure 7. A schematic diagram showing the breakage of the starting and ending positions of the inversion variation on chromosome 6 of MH63. "Reference" represents the sequence of chromosome 6, and "query" represents the sequence of contigs after breakage.

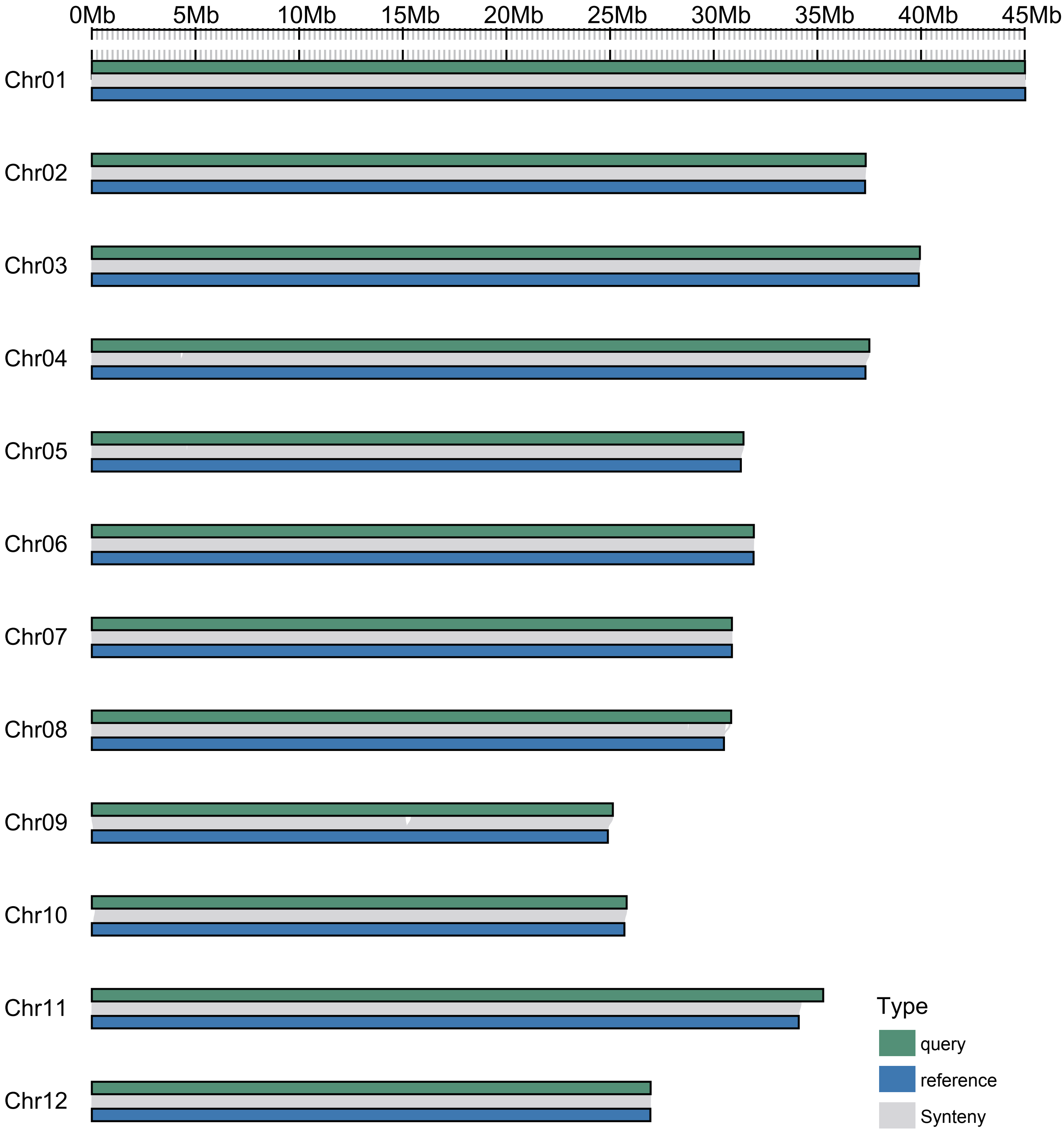

Additional file 1: Figure 8. The collinearity map between RAGA-optimized assembly and original MH63 T2T genome indicates complete consistency on chromosome 6.

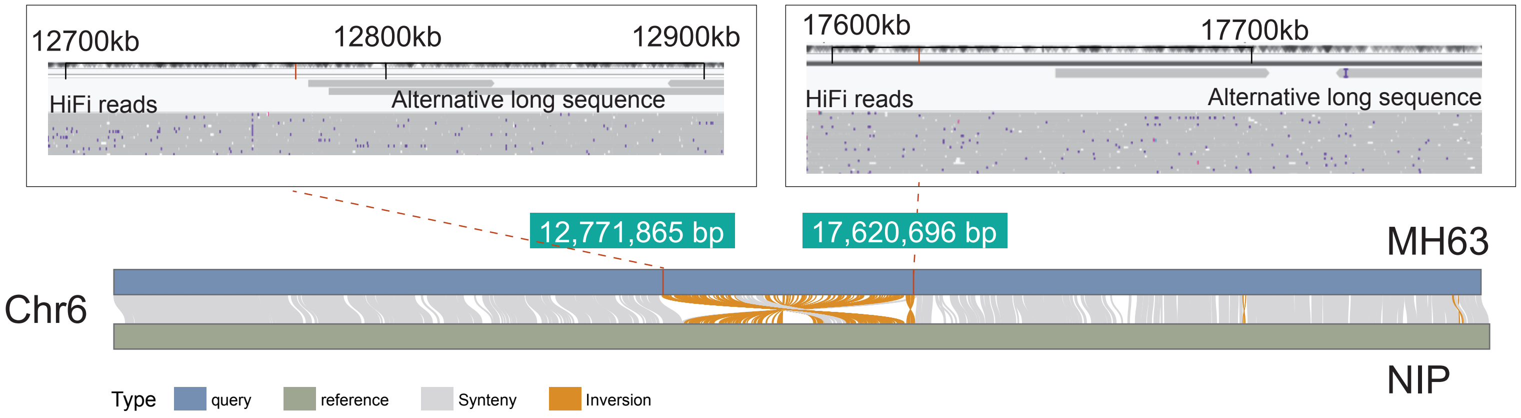

Additional file 1: Figure 9. IGV re-alignment maps after comparing the alternative long sequences generated by RAGA and PacBio HiFi reads with the MH63 T2T reference genome. It was found that RAGA did not generate sequences spanning the inversion breakpoints.

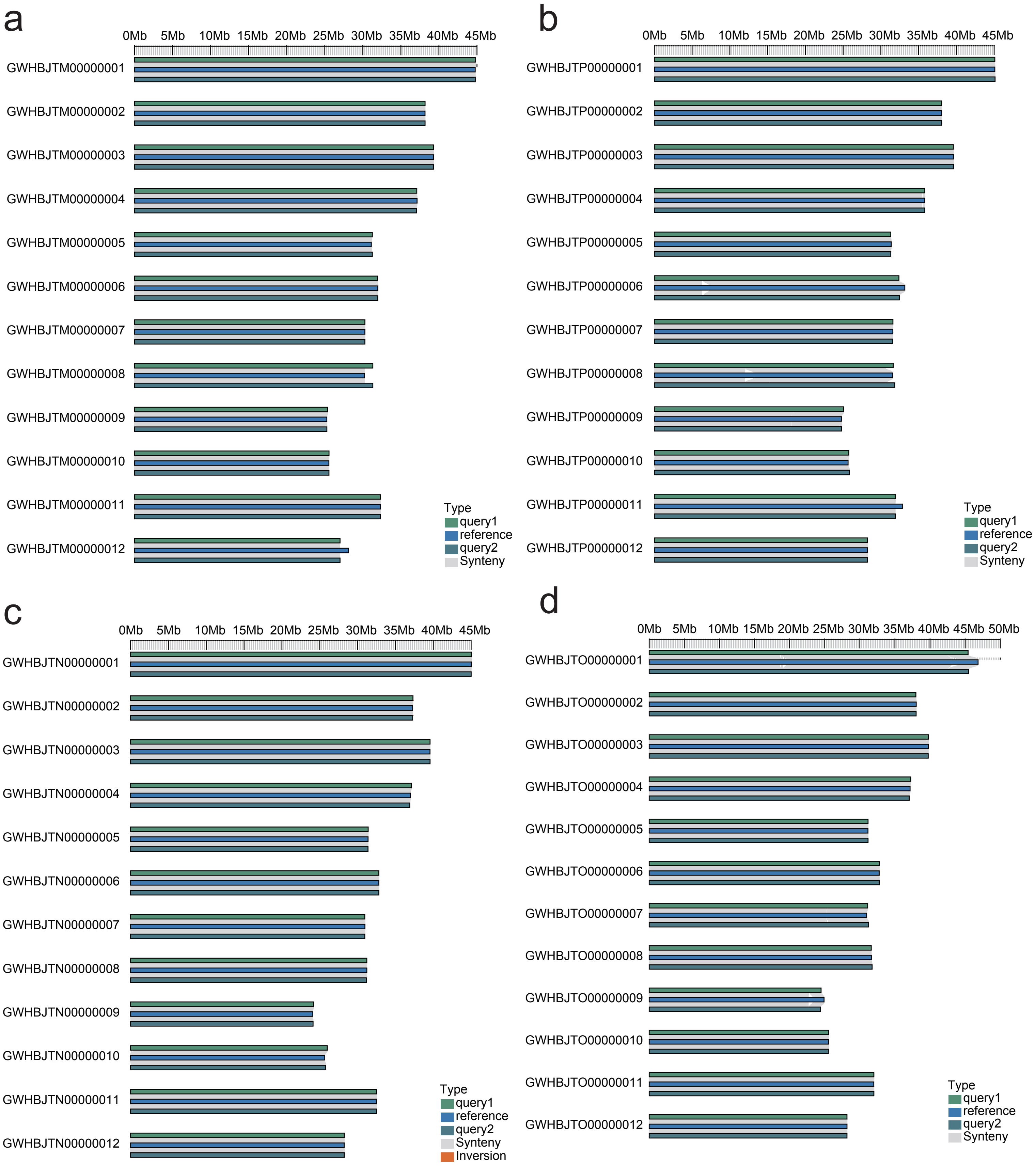

Additional file 1: Figure 10. The collinearity graph shows the collinearity between *de novo* assembly, reference T2T genome and RAGA-optimized assembly. In the graph, "query1" represents *de novo* assembly, "reference" represents reference T2T genome, and "query2" represents RAGA-optimized assembly. **(a)**, HuaZhan; **(b)**, J4155S; **(c)**, LK638S; **(d)**, XL628S.

gap1: Chr07:29,799,587-29,825,302

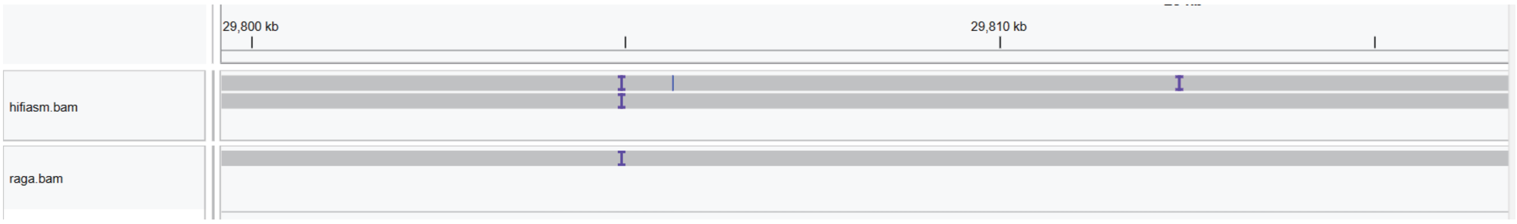

gap2: Chr08:28,752,587-28,768,698

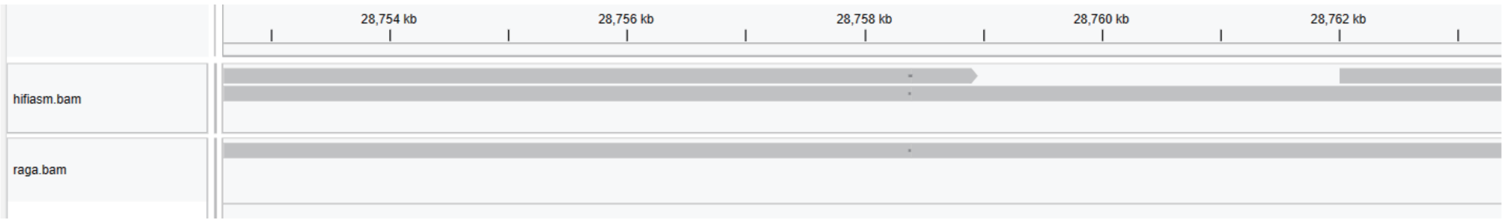

gap3: Chr08:28,774,179-28,790,290

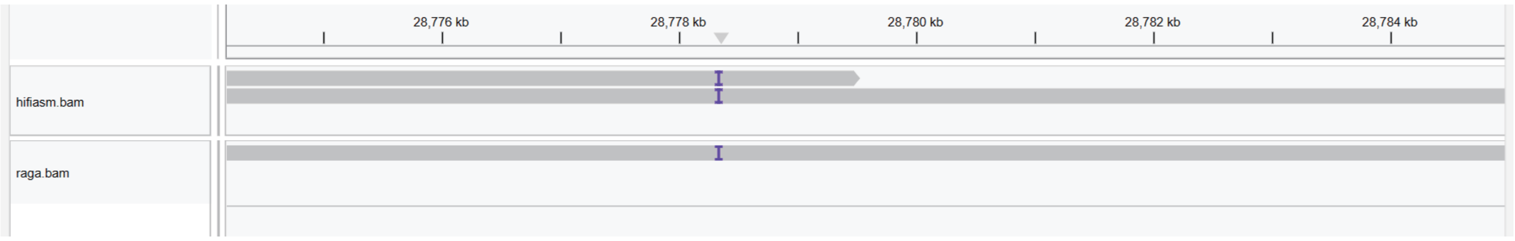

Additional file 1: Figure 11. The alignment of *de novo* assembly, RAGA-optimized assembly with the MH63 T2T reference genome near gap regions in Figure 3.

ptg000001l:917,187-1,003,325

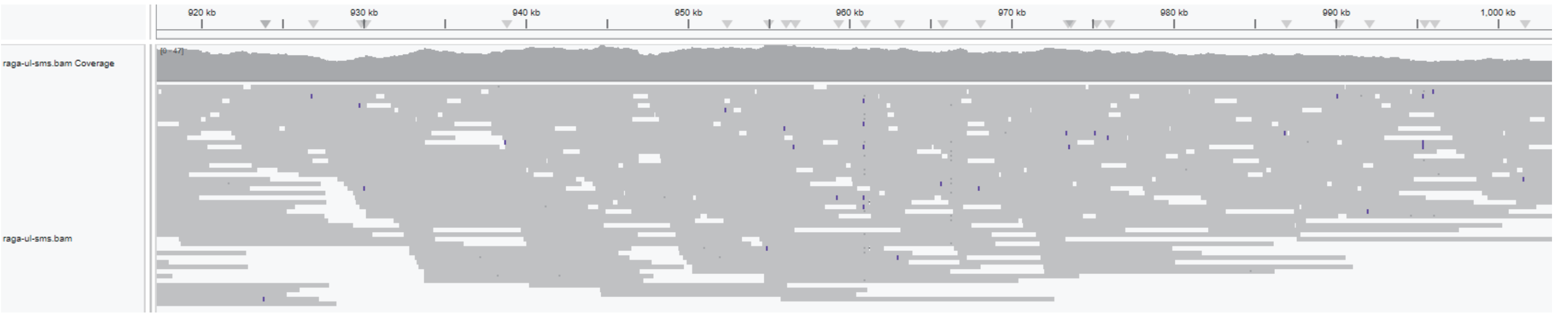

Additional file 1: Figure 12. Use IGV to perform PacBio HiFi reads alignment inspection on the regions composed of alternative long sequences in the RAGA-optimized assembly of ZH13.
